# Population epidemiological study on the prevalence of dizziness in the city of São Paulo

**DOI:** 10.5935/1808-8694.20130127

**Published:** 2015-10-08

**Authors:** Roseli Saraiva Moreira Bittar, Jeanne Oiticica, Marco Aurélio Bottino, Fernando Freitas Ganança, Riva Dimitrov

**Affiliations:** a PhD in Medicine; Assistant Doctor at the Neurotology Department HCFMUSP; b PhD in Medicine; Adjunct Professor of Otology and Neurotology at UNIFESP (Paulista School of Medicine - Federal University of São Paulo); c MSc in Medicine. Medical School of the University of São Paulo

**Keywords:** dizziness, epidemiology, prevalence

## Abstract

The epidemiology of dizziness is essential in clinical practice.

**Objective:**

To establish the prevalence of dizziness in the adult population of São Paulo, its clinical characteristics and level of discomfort.

**Method:**

A prospective cross-sectional study ran from April to October of 2012 by a field questionnaire, totaling 1,960 interviews. The predictor variables assessed were age, gender, type of dizziness and the dizziness disability index. The statistical tools used to assess the significance between variables were the chi-square test, *Student's* t-test and logistic regression. We used a 95% confidence interval for estimated values.

**Results:**

The prevalence of dizziness in the city of São Paulo was established at 42%. We found two peak of complaints, 49% in the range of 46–55 years and 44% in the elderly. Vestibular-related dizziness was estimated to affect 8.3% of the population, mainly women (*p* < 0.001). The symptoms caused disability in 27% of symptomatic interviewees and it is more bothersome to females (*p* < 0.001), who more frequently seek medical care (*p* < 0.001).

**Conclusion:**

The prevalence of dizziness in São Paulo was found to be 42%. It affects daily activities in 67% of symptomatic patients, but only 46% of them seek medical help.

## INTRODUCTION

Body balance is fundamental in the life of the individual and it causes high anxiety to patients; gait difficulties; disorientation; social exclusion and isolation1., 2., 3., 4.. According to the Committee on Hearing and Equilibrium of the American Academy of Otolaryngology and Head and Neck, dizziness can be defined as any illusory sense of motion without any real movement in relation to gravity^5^. The symptom may or may not have originated on the vestibular system, and it comprises sensations described in many different ways: dizziness (vertigo), imbalance, instability or fluctuation, pre-syncope or syncope, motion sickness, oscillopsia and falls. It happens at any age, from the first months of life all the way to old age2., 6., 7., 8., 9.. Non-vestibular dizziness are often poorly defined, usually described as malaise, dizziness, lightheadedness, “feeling faint”. They often represent symptoms of poor central nervous system perfusion and/or they may be associated with heart problems, postural hypotension, ischemic episodes or metabolism disorders^10^. Central origin dizziness is often more insidious and less intense than those of vestibular origin.

Dizziness epidemiology is of fundamental interest in clinical practice, especially among neurologists and otolaryngologists because the symptom is one of the 10 leading causes of visits to emergency services11., 12.. It is estimated that 20% of patients seeking a general practitioner have some kind of dizziness13., 14.; the third most common clinical complaint in a general clinic, second only to pain and fatigue^15^. Dizziness prevails in females (1.3 to 1) and its incidence increases directly with age, peaking between 65 and 75 years16., 17.. A large percentage of dizziness spells are benign and undergoes natural compensation18., 19.; however, it may be the first symptom of more severe cases, such as stroke or tumors.

Numerous studies have shown that dizziness is one of the most prevalent complaints in medicine, affecting approximately 20% to 30% of the world population14., 20., 21.. However, its high prevalence in population surveys may be influenced by methodological biases, including how the questionnaires are applied, by conventional mail or Internet, or in how the symptom is described. A large epidemiological study recently conducted in Germany combined screening a large and representative population sample of 4,869 individuals with moderate to severe dizziness or vertigo, with the validation of these complaints through consultation with neurotologists in 1,003 of these individuals. The prevalence of vestibular-borne dizziness established for adults with ages 18 to 79 years was estimated at 7.4% (95% CI: 6.5 to 8.3%), and the frequency was three times greater in the elderly than in young adults^17^. The study also showed that vertigo is recurrent (88%) and causes severe problems (80%) in most cases, with interruption of daily activities, leave of absence and frequent medical appointments17., 22..

According to the latest IBGE census of 2010, the city of São Paulo has a population of 11,253,503 inhabitants. Its birth rate is 15.59/1,000, the elderly representing 12.53% of the population and an illiteracy rate of 3.18%. The annual per capita income is R$ 39,445.20, and 58.40% of the population graduate from high school^23^. These sociodemographic characteristics provide the city of São Paulo with the same profile as the majority of European countries. The demographic transition process seen with the fertility decline and increased survival, directly influences the age structure of the population, which transfers to the elderly the responsibility for supporting themselves economically. Thus, the social demands of São Paulo have been changing and it becomes necessary to better understand this population so that we can develop more adequate healthcare policies^24^.

The public and private healthcare networks in the city of São Paulo have no data on the prevalence of dizziness. Thus, compilation, analysis and communication of such information may help outline the profile of the population. It starts with the assumption that the dizziness is a prevalent complaint in São Paulo, with potential morbidity and impact on people's health, particularly in the elderly. Thus, additional information about the frequency, determinants and associated comorbidities may improve primary care and guidance of patients in screening areas, in addition to provide foundations for public health policies and prevention campaigns. Moreover, risk factors collected from population samples may raise new pathophysiological hypotheses and help us advance in the treatment of dizziness. Determining the prevalence of dizziness will meet the need to know this information in the municipality, in order to implement projects involving the prevention and rehabilitation of the target population. Without a real estimate, it is impossible to plan any kind of action, including obtaining resources or support for these projects.

This epidemiological survey aimed at:

1. Establishing the prevalence of dizziness in the adult population of São Paulo;
2. Describing the main clinical characteristics of dizziness in this population;
3. Quantifying the degree of annoyance the symptom caused to the population.

## METHODS

This cross-sectional study was carried out between April and October of 2012, in São Paulo, which has a population of approximately 11,253,503 inhabitants according to the most recent IBGE census of 2010^23^. The population survey used to determine the prevalence of dizziness in the city of São Paulo was conducted through a questionnaire to the population, previously created for this purpose; adapted and modified from the original study to determine the prevalence of Meniere's disease in the population of southwestern Finland^21^ and Disability Index^25^. The project was conducted in accordance with the standards and guidelines established by the ESOMAR International Code of Practice for Social Research and Marketing, and after approval by the Ethics Committee for Analysis of Research Projects, research protocol #: 0970/09. The project received funding from the Brazilian Association of Otorhinolaryngology and Facial and Neck Surgery (ABORL-CCF), through the bidding process used to grant scholarships for epidemiological projects and also the Foundation for Research Support of the State of São Paulo (FAPESP/Regular Support to Research/Process #: 2011/10343-7).

### Sample

Based on population studies, the sample size was calculated assuming an expected 10% prevalence of dizziness in the population, with an accuracy of 2%, confidence interval of 95%, design effect of 2, and 10% increase for possible losses. The initial sample size was calculated at 1,901 inhabitants. Estimating 3–4 people per household, for a total estimated number of 633 households visited. It is expected that 40 of the 13,193 census districts in São Paulo were randomly selected, eight in each of the five regions of the city (north, south, east, west and center). The five regions were included to ensure sample diversity, thus ensuring an estimate for all walks of life and age groups. The random selection of the census district was proportional to the population size of the region, to maintain equal likelihood.

### Data collection

We used cluster sampling among the different census districts. In order to select the households to be visited for data collection, we randomly selected the census districts. Within each of those districts, we randomly selected one block and a corner of this block. From this corner selected, we consecutively visited the first 16 homes. A specialized company, with large experience in this field - Analítica Pesquisas Mercadológicas, Sociais e Econômicas Ltda - every adaptation and encoding of the population structured questionnaire; pre-tests; prepared the cards; field manual and control material; team training; random selection of the census districts; fieldwork; personal interviews at home and statistical analysis of the results collected.

### Inclusion Criteria

We included individuals aged 18 years and of both genders. In the population sample, all dwellers of each household selected were interviewed, after agreeing with the terms and signing the consent form. When there were two or more families in the same plot, each was considered separately.

### Exclusion Criteria

We used the following exclusion criteria vis-à -vis the homes selected: (1) residents who were not home after three attempted visits, (2) those who were sick and recovering, (3) non-residents in the home that was being visited among them the relatives and/or friends and maids who do not dwell in the house, (4) commercial houses where no one lived and empty houses.

### Study variables

The extent of occurrence was evaluated by the ratio between the number of individuals with dizziness in relation to the total number of interviewees. The main predictor variables evaluated were gender, age, education, occupation, race, defined as qualitative variables. Quantitative variables included the degree of dizziness discomfort as measured by the Disability Index^25^.

### Statistical Analysis

The variables investigated were subjected to descriptive analysis. The significance of the association between qualitative variables and the extent of occurrence, presence of dizziness, was established by the chi-square (*x*^2^). For quantitative variables we employed the *Student's t*-test. The variables that showed significant association with the extent of occurrence (*p* less than 0.05) were subjected to a logistic regression model to identify possible confounders of the association and to identify the factors most strongly associated with the presence and severity of dizziness. We also calculated the 95% confidence intervals for the estimates produced (e.g. prevalence of dizziness).

## RESULTS

### Sample

The company responsible for the field survey chose to expand the sample size initially calculated at the time of collection, to reduce possible losses. Therefore, our final sample consisted of 1,960 individuals. To obtain this number, 1,008 households were visited, 63 randomly drawn among the 13,193 census districts in São Paulo, which accounted for an average of 2.2 adults per household (Table 1).

**Table 1:** Sample size (n) and percentage (%) of households visited according to the census of the city of São Paulo.

Areas	Total	
	n	%
East	738	38
South	589	30
North	305	16
West	194	10
Center	134	7
Sample Base	1960	100

n: Sample size; % Percentage of the sample.

The descriptive analysis of the main predictor variables assessed (gender, age, education, occupation, race) is itemized on Table 2.

**Table 2:** Descriptive analysis of the profile of the sample collected for the city of São Paulo.

		n	%
Gender	Females	1046	53
	Males	914	47
	18/25	278	14
	26/35	404	21
	36/45	349	18
Age	46/55	325	17
	56/65	273	14
	66 e +	328	17
	Refusal	3	*
	Illiterate/Incomplete basic education	292	15
	Complete basic education/Incomplete junior high school	484	25
Schooling	Complete junior high school/Incomplete senior high school	346	18
	Complete senior high school/Incomplete higher education	620	32
	Complete higher education	218	11
	Whites	1237	63
	Blacks	167	9
Race (Per observation)	YellowsBrowns	45507	226
	Indians	4	*
Sample Base		1960	100

n: Sample size; % Percentage of the sample

* Percentage value lower than 0.003%.

### Prevalence of symptoms in the population of the city of São Paulo

#### Dizzy feeling

The “dizzy feeling” complaint prevalence among the population of the city of São Paulo was 42% (831 subjects) versus 58% (1,129 individuals) who denied this complaint. The symptom affects a greater percentage of women (52%) compared to men (31%). Application of the Person's chi-square test revealed statistical significance (*p* = 0.048) in the evaluation of dizziness reported by the age groups. The age-wise trend is one of fluctuation with complaint peak (49%) in the range of 46–55 years (Table 3).

**Table 3:** The “dizzy feeling” complaint prevalence according to the various age groups in the population of the city of São Paulo.

							Age							
Have you ever felt dizzy?	Total		18 to 25 years		26 to 35 years		36 to 45 years		46 to 55 years		56 to 65 years		Over 65 years	
	n	%	n	%	n	%	n	%	n	%	n	%	n	%
Yes	831	42	117	42	166	41	128	37	158	49	118	43	144	44
No	1129	58	161	58	238	59	221	63	167	51	155	57	184	56
Sample Base	1960		278		404		349		325		273		328	

Chi-squared test			
	Value	DF	*P*
Pearson's chi-squared	12.705a	6	0,048
Odds ratio	13,822	6	0,032
Linear by linear association	1,275	1	0,259

n: Sample size; % Percentage of the sample; DF: Difference; *p*: Statistical significance (two-tails); a: 2 cells (14.3%) expected count less than 5. The minimum expected count is 1.27.

### Dizziness description

To better organize the answers in item: “how would you describe your dizziness” we have a list containing four possible descriptions of the symptom for the respondent to indicate the phrase that best defines his/her feeling. The most common and most often stated was vertigo (a feeling that either the body or the environment is turning), in 41% of the 831 subjects asked. Second, floating (sensation of lightheadedness, “void head”, heavy head), stated by 27% of respondents. Thirdly, fainting sensation (feeling of impending fainting, usually with vision loss or vision blurring), with 18% prevalence. And finally, imbalance (difficulty in maintaining posture engaging the body), reported in 14% of the cases. The “imbalance” complaint, reported by 14% of the symptomatic population, increased to 24% in the range of more than 65 years, demonstrating a significant increase (*p* = 0.017, Pearson's Chi-square test) of prevalence with age (Table 4). Table 4 is the type of dizziness in relation to age. Vertigo is more prevalent in the younger age group (reaching 49% of respondents aged between 18 and 25 years). The imbalance grows continuously with age, peaking in individuals over 65 years.

**Table 4:** Distribution of the various descriptions of the “feeling dizzy” complaint (n = 831) according to age groups in the population of the city of São Paulo.

How would you Age describe your dizziness?	Total		Age											
			18 to 25 Years		26 to 35 Years		36 to 45 Years		46 to 55 Years		56 to 65 Years		Over 65 Years	
	n	%	n	%	n	%	n	%	n	%	n	%	n	%
Vertigo	343	41	57	49	78	47	46	36	58	37	50	42	54	38
Fainting	146	18	21	18	33	20	28	22	28	18	20	17	16	11
Unbalance	114	14	10	9	15	9	14	11	24	15	17	14	34	24
Fluctuation	228	27	29	25	40	24	40	31	48	30	31	26	40	28
Reduced Base^*^	831	117	166	128	158	118	144							

Chi-square test			
	Value	DF	*P*
Pearson's chi-square	28,842a	15	0,017
Odds ratio	28,107	15	0,021
Liner by linear association	5,673	1	0,017

n: Sample size; % Sample percentage; *Individuals with dizzy feeling; DF: Difference; p: Statistical significance (two-tailed); the: 0 cells (0%) expected count less than 5. The minimum expected count is 16.05.

### Dizziness-related nausea

**Graph 1:**
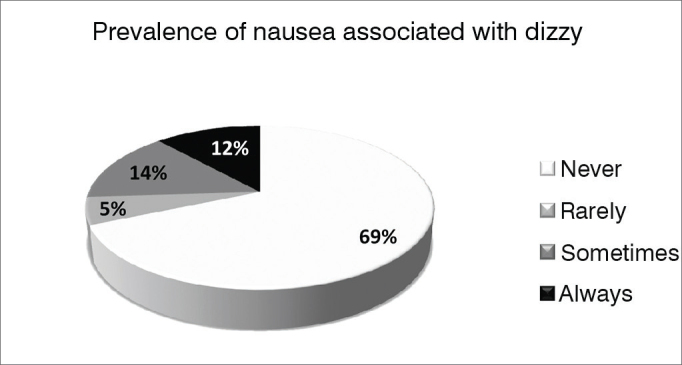
Prevalence of nausea associated with “feeling dizzy” (n = 831) in the population of the city of São Paulo.

Most respondents (69%), never had nausea associated with “feeling dizzy”. The distribution of responses can be seen in Graph 1.

Analyzing the subgroups, 83% of men reported never having nausea-related dizziness, against 61% of women (*p* < 0.001, chi-square test). With respect to age, absence of nausea associated with dizziness tended to be more prevalent (79%) in young individuals - aged 18–25 years, despite the fact that the difference between age groups was not statistically significant (*p* = 0.110, Pearson's chi- square test).

Frequency at which of dizziness happens

**Graph 2:**
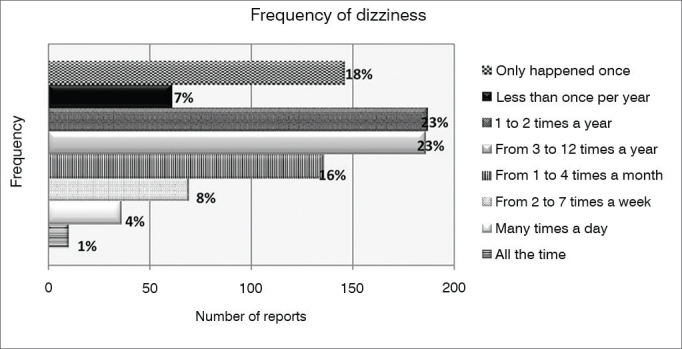
Distribution of the frequency of occurrence of symptoms among those who report “feeling dizzy” (n = 831) in the population of the city of São Paulo.

To better organize the answers to this item, we presented a list of eight possible rate of symptom occurrence, for the respondent to indicate the one that best describe his/her situation. Graph 2 depicts the distribution of responses.

The reported incidents of minor importance with respect to the frequency (it only happened once or less than once per year) were mentioned by 18% and 7% of respondents, respectively. Most respondents (46%) reported having symptoms one to 12 times per year. As the frequency of dizziness increases, the number of cases decreases, until reaching a frequency of 1% of respondents who stated feeling dizzy all the time. As far as the subgroups are concerned, the frequency tended to fluctuate much, but we noticed that among the younger, “feeling dizzy” tends to be less frequent than among the elderly. This difference was statistically significant (*p* < 0.001, chi-square test). In addition, all age groups had dizziness spells peaking between 1x per year and 1x per month.

### How long they had been feeling dizzy

The analysis of the item: “how long have you been feeling dizzy” (question 21 in Annex 1), had a large variation in disease progression time as per reported by the respondents. Reports ranged from less than one year (a few months) to over 30 years; this variability hampered the search for an analysis parameter. We have compiled the responses into time slots that helped us better display the content (Table 5). We noticed that, when applied to age groups, the chi- square test was statistically significant (*p* = 0.001) vis-à -vis the results (Table 5), with higher prevalence of long standing dizziness among older individuals, when compared to younger patients. We also realized that more than half of the patients (59%) had been having dizziness spells between zero and three years.

**Table 5:** Distribution of “feeling dizzy” complaint along time (n = 831) according to the different age groups in the population of the city of São Paulo.

For how long have you been feeling dizzy?	Total		Age											
			18 to 25 Years		26 to 35 Years		36 to 45 Years		46 to 55 Years		56 to 65 Years		Over 65 Years	
	n	%	n	%	n	%	n	%	n	%	n	%	n	%
Up to 1 year	255	31	47	40	65	39	37	29	43	27	35	30	28	19
> 1 up to 3 years	232	28	38	32	50	30	42	33	36	23	32	27	34	24
> 3 up to 5 years	101	12	12	10	20	12	14	11	23	15	15	13	17	12
> 5 up to 10 years	130	16	14	12	14	8	21	16	27	17	18	15	36	25
> 10 up to 20 years	66	8	4	3	13	8	8	6	16	10	9	8	16	11
> 20 up to 30 years	29	3	2	2	1	1	6	5	8	5	4	3	8	6
> 30 years	7	1	-	-	-	-	-	-	4	3	3	3	-	-
Refused	1	^*^	-	-	-	-	-	-	-	-	-	-	1	1
Does not know/remember	10	1	-	-	3	2	-	-	1	1	2	2	4	3
Reduced Base^*^	831		117		166		128		158		118		144	

Chi-square test			
	Valor	DF	*P*
Pearson's chi-square	319,493a	245	0,001
Odds ratio	322,963	245	0,001
Linear by linear association	2,954	1	0,086

n: Sample size; % Percentage of the sample; ^*^ Individuals feeling dizzy, DF: Difference, *p*: Statistical significance (two-tailed), to: 261 cells (87%) expect to count less than 5. The minimum expected count is 0.14.

### Level of disability caused by dizziness

In order to investigate the level of discomfort caused by dizziness, we presented the respondent with a list of six possible descriptions, based on the Disability Index^25^, so that he/she could indicate the phrase that best described his/her situation. The first two sentences (1 = symptoms are negligible, do not bother and 2 = symptoms bother you, but without causing disability) were the most frequently mentioned, by 33% and 40% of respondents, respectively. The third sentence (the symptoms bother you, cause mild disability, you do the usual tasks (work, home), but the symptoms interfere with activities outside the home), which describes mild disability, was reported by 14% of respondents. The fourth phrase (the symptoms bother you, cause moderate disability, interference with usual tasks and those outside the home), indicating moderate disability, was mentioned by 11% of respondents. The last two sentences, which report more severe discomfort with severe recent disability (symptoms bother, there is recent severe disability, he/she is, or has been on sick leave or had to change jobs because of symptoms) or long duration (the symptoms bother, there is severe and longstanding disability, the person is unable to work for more than one year or is on permanent leave), had much lower prevalence in the sample, 1% and 2%, respectively. With regard to gender, 44% of men reported that symptoms do not bother against 27% of women, a statistically significant difference (*p* < 0.001), Pearson's chi-square test).

### Medical consultation because of dizziness

Just under half (46%) of those who reported dizziness (n = 831) had visited a doctor for this reason, while 54% of respondents had never sought the doctor because of this complaint. When analyzing the subgroups, we realized that women (51%) more often than men (37%) sought medical help for the treatment of symptoms, with a statistically significant difference (*p* < 0.001, chi-square test). On the issue of age, older people most often seek medical help for dizziness than younger ones (Table 6).

**Table 6:** Medical consultation for complaints of “feeling dizzy” (n = 831) according to age groups in the population of the city of São Paulo.

Have You Been To The Doctor Because Of Dizziness?	Total		Age											
			18 to 25 Years		26 to 35 Years		36 to 45 Years		46 to 55 Years		56 to 65 Years		Over 65 Years	
	n	%	n	%	n	%	n	%	n	%	n	%	n	%
Yes	382	46	32	27	62	37	47	37	87	55	66	56	88	61
No	449	54	85	73	104	63	81	63	71	45	52	44	56	39
Reduced Base^*^	831		117		166		128		158		118		144	

Chi-square test			
	Value	DF	*P*
Pearson'schi-square	48,975^a^	5	0,000
Odds ratio	49,840	5	0,000
Linear by linear association	44,610	1	0,000

n: Sample size; % Percentage of the sample; ^*^ Individuals feeling dizzy, DF: Difference, p: Statistical significance (two-tailed), ^a^: 0 cells (0%) expected count less than 5. The minimum expected count is 53.78.

## DISCUSSION

Dizziness is a subjective and nonspecific symptom that can only be properly diagnosed by clinical examination. Thus, we must consider that the judgment subjectivity of respondents as well as the temporality of complaints reported, may, by themselves, be considered bias elements. Do you have or have had dizziness? “Yes” was answered by 42% (831 subjects) versus 58% (1,129 individuals) of respondents who denied the symptom. If we excluded individuals who reported having had only one episode of dizziness in the past, this percentage drops to 41% (812 individuals). Dizziness is one of the most common and prevalent complaint in clinical practice, affecting approximately 20% to 30% of the general population, considering the various epidemiological studies published^17^. The rate found in our study is high when compared with other studies published, such as Kroenke et al.^26^, describing 23.2% prevalence of symptoms in a population study which objective was to seek psychosomatic symptoms. Yardley et al.^14^, found a 23.3% prevalence of dizziness researching British workers by mail, or even those of Neuhauser et al.^27^, who interviewed 8,318 adults by telephone and reported a 29.5% prevalence of dizziness or moderate to severe vertigo, presumably of vestibular origin. On the other hand, another prevalence study was conducted in rural India and the estimated index of “dizziness” was 0.71%. Therefore, the observed difference in the prevalence of “dizziness” among the various population studies published is related not only to how data is collected, further still with the standards considered in their analysis. Not only do the results differ widely, but they also refer to specific groups in which the main objective was not to directly assess dizziness in the general population. Other studies carried out in healthcare services can not be extrapolated to the population, as well, since there was a sample selection bias28., 29.. Thus, dizziness proved to be a highly prevalent symptom in the population of the city of São Paulo.

In our sample, women (52%) were more often affected than men (31%), at a ratio of 1.67 to 1, confirming reports from the world literature14., 16., 17., 26.. We believe that the relative higher prevalence in women can be attributed to factors such as hormonal cycle variation^30^, higher prevalence of migraine^22^ and the fact that women seek more medical care. Our series showed that women (51%) seek medical attention more frequently than men (37%) because of dizziness. However, seeking more treatment is not the reason why the symptom were more common in females, since respondents were randomly chosen. We believe that a woman's monthly hormonal variation is actually the trigger for migraine and dizziness episodes in the premenstrual period, as well as the use of hormones for contraceptive purposes. And yet, the menopause period has dizziness as one of its main symptoms^30^.

There was statistical significance (*p* = 0.048) when we assessed the presence of dizziness by age groups. According to the literature, the prevalence of dizziness increases slightly with age in the community, but markedly in medical practice^28^. In our survey, the trend through the different ages oscillated with the peak of the complaint (49%) in the range of 46–55 years. It is interesting to note that this is the age group women in menopause women, with migraine peaks31., 32. and variation in metabolic hormonal profile^30^. The specialists in this field also know that a significant proportion of cases that visit the doctor with complaints of dizziness consists exactly of women in this age group. Therefore, the sample obtained reinforces the idea of the impact of menopause on the quality of life of these patients.

The build up of comorbidities such as cardiovascular and neurological diseases associated with balance system aging are proposed etiologies for dizziness in the subjects over 60 years^33^. In our series, the elderly accounted for the second peak (44%) in the frequency of dizziness complaints. In Brazil, the prevalence of dizziness in this age group is estimated at around 45%^34^. According to reports from the world literature, the prevalence of dizziness in the elderly population reaches 41.4% when considering all cases, including having had only one previous episode; but it falls to 9.6% when the analysis is restricted to regular dizziness episodes (frequency of at least once a month)^35^. In approximately 20% of the elderly affected by dizziness, the symptom is serious enough to jeopardize their daily life activities^36^. Our experience indicates that 44% (144 individuals) of the population over age 65 have the symptom and 68% of these symptoms do not cause disability; 17% had mild disability and in 15% the symptoms interfere with usual activities or those carried out outside the home. Therefore, our data confirms the literature data.

Regarding the type of dizziness described by respondents, the highest prevalence of vertigo was found in the younger age group and reaches 49% (57 individuals) between 18 and 25 years and 47% (78 individuals) of individuals between 26 and 35 years, suggesting that the younger population is more often affected by peripheral dizziness. Research has shown that diet errors (excess sugar, fasting, high fat consumption, etc.) and social activities (consumption of licit or illicit drugs, irregular sleep routine, etc.) are very much present today37., 38. and migraine triggers would be responsible for triggering dizziness^39^. However, Dizziness is clinically characterized as vertigo accompanied by nausea, vomiting, positional vertigo and oscillopsia and its prevalence was determined to be 7.4% for adults aged between 18 and 79 years^17^. In our survey, dizziness described as vertigo was present in 41% (343 individuals) of 831 subjects who reported “feeling dizzy”, corresponding to 17% of the general population. Also, in our sample, vertigo was usually accompanied by nausea in 26% (163) of the subjects, corresponding to 8.3% of the sample population investigated. This fraction of the population, which describes the dizziness of very likely being of vestibular origin, which is quite similar to the rate found in Germany by Neuhausen et al.^17^, who estimated their frequency of vertigo as being three times higher in the elderly. We did not observe this higher prevalence of dizziness in the elderly. The percentages of the symptom “dizziness” increased slightly with age, 20% between 18 and 25, to 32% between 56–64 years, falling to 21% from 65 years of age.

However, it is interesting to notice that in our sample the elderly had a higher prevalence of imbalance in relation to the rest of the population. Imbalance-type dizziness had proportionally higher prevalence with age until it reaches 24% of symptomatic elderly (*p* = 0.017). This observation is in agreement with the clinical characteristics of human balance system aging plus the comorbidities that build up along one's life^7^. Symptoms such as fluctuation (27% of respondents) and presyncope (18% of respondents) are dizziness of systemic and/or central characteristics and affected the different age groups in a similar fashion.

When present, dizziness spells tend to be episodic with frequency ranging from once a year to once per week (61% - 509 individuals). The daily and persistent symptom affects 3% (five subjects) of symptomatic individuals and characterize non-peripheral etiologies such as the use of medication, CNS lesions or psychiatric background^36^. We could not establish statistical significance between age groups, since the frequencies varied greatly between age groups. The time of dizziness evolution was also very variable, from a few months to 30 years, with an average between zero and three years (59% of symptomatic) and showed statistical significance between age groups. The vast majority of symptomatic individuals (69%) had the symptoms for over a year. The longer complaints were found in older individuals compared to younger ones (*p* = 0.001). This significance is easily explained by the symptom duration over time, affecting more the older individuals.

Dizziness proved to be a highly prevalent occurrence in the population, resulting in various levels of disability. When we asked the questions regarding the content of incapacity caused by dizziness, women (73%) were more annoyed than men (56%). The dizziness-caused disability was present in 67% of symptomatic respondents. Among them, 40% of subjects reported discomfort without activity limitations, and interference of symptoms on daily activities were reported by 27% of subjects. These indices characterize dizziness as a symptom that interferes with the quality of life of individuals and their work activities. According to the literature, 8% of affected individuals interrupt their activities due to the symptom^40^. Although most subjects are able to perform their routine activities, these patients feel very uncomfortable and unsafe. Because it is subjective and does not prevent the realization of these activities, the symptom is often underestimated by the family and by the physician, marginalizing the patient and causing mental illnesses such as anxiety and depression^40^.

More important is the fact that the dizziness affect a large portion of the population, clearly impacting their quality of life, and yet, more than half of those affected (54%) did not seek medical attention. Women (51%) sought medical help more often than men (37%), a statistically significant difference *(p* < 0.001). In terms of age, we observed that the demand for assistance increases in direct relation with age; and from 46 years, that percentage surpasses 50%, affecting 61% of the elderly. This fact is possibly associated with the minor importance youngster attach to their symptoms. Going to the doctor is directly proportional to the degree of discomfort caused, naturally by affecting the individual's day-to-day activities.

The impact “dizziness” has been underestimated by health professionals over the years. Its high prevalence in the population implies the need for public policies that have the purpose of viewing it as a primary public health issue. We should also draw attention to the fact that associated mental disorders increase misuse and meaningless use of healthcare services. Few of these individuals have access to proper care by mental health workers.

## CONCLUSION

Dizziness proved to be a highly prevalent symptom in the city of São Paulo and affects 42% of its adult population. It affects mostly women, at a ratio of 1.67 to 1 male subject. There were two prevalence peaks, the first between 46 and 55 years and the second in the elderly. Vertigo was more frequent in the younger age group and balance disorders increase in direct relation with age. Vertigo accompanied by nausea, which characterizes vestibular-related dizziness comprised 8.3% of the population and was found in similar proportions in different age groups. The vast majority of individuals had had symptoms for over a year. Women feel more disabled than men and look for help in medical services more often. Dizziness interferes with daily activities in 67% of symptomatic respondents; however, there is a significant difference with regards to gender, with women feeling more annoyed than men. Despite this, only 46% of respondents sought medical help, and this frequency of medical consultation by dizziness is higher among women and the elderly.

## FINANCIAL SUPPORT

Brazilian Association of Otorhinolaryngology and Facial and Neck Surgery (ABORL-CCF), under the administration of Prof. Ricardo Ferreira Bento - bid for research support grants and epidemiological projects.

Foundation for Research Support of the State of São Paulo (FAPESP)/Regular Research Support/Process #: 2011/10343-7.
